# Malik Hussain Mubbashar, MB BS, FRCP (London), FRCP (Edinburgh), MRCP (Glasgow), FCPS (Pakistan), FRCPsych

**DOI:** 10.1192/bjb.2021.28

**Published:** 2021-06

**Authors:** David P. Goldberg, Fareed A. Minhas

Formerly Professor and Head of the Institute of Psychiatry, Rawalpindi, Principal, Rawalpindi Medical College and Vice-Chancellor, University of Health Sciences, Lahore, Pakistan

**Figure:**
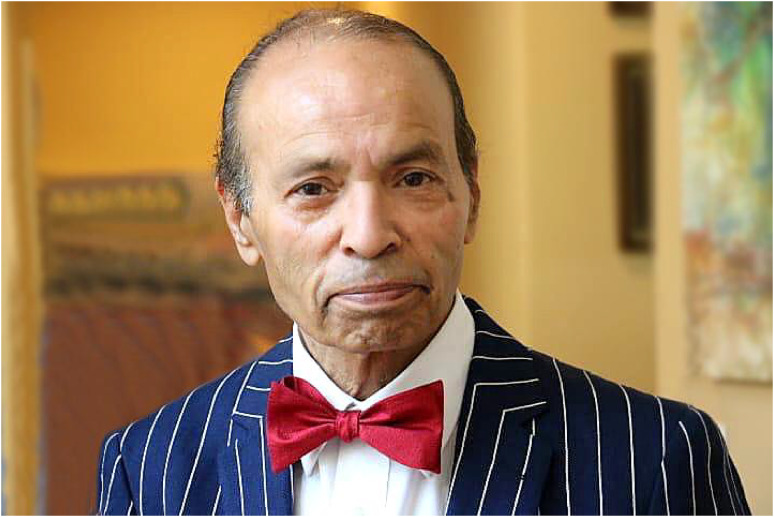

Malik Hussain Mubbashar, the leader of psychiatry in Pakistan for a generation and a pioneer in the development of community mental health services and psychiatric research, died on 10 August 2020, at the age of 75 years. His work in the establishment of community mental health services served as a model for such services in low-income countries throughout the world. Initially, local men were recruited and trained to provide services to those identified in need. This was soon followed by school programmes, which enlisted head teachers, persuading them to include information about mental disorders in their curricula. Professor Mubasshar was remarkably innovative in his approach, for example, recruiting children to identify people in their village who might benefit from seeing a doctor and arranging travel for them. His research activities were always both scientifically rigorous and highly relevant to the needs of people with mental health problems in areas where specialist services were thin on the ground or non-existent. He carried out a community survey of stress and psychiatric disorder in rural Punjab, a prevalence study of psychiatric morbidity among the attendees of a native healer in Rawalpindi, as well as a unique randomised trial of the impact of a school mental health programme in rural Rawalpindi, Pakistan. He had a highly productive publication record, authoring or co-authoring many peer-reviewed papers as well as 28 books.

He was highly effective as a teacher. At the Rawalpindi Medical College (RMC), where he was Principal for some years, he reorganised undergraduate education, introducing problem-based learning. As Vice-Chancellor of the University of Health Sciences, Lahore, he made the study of behavioural sciences mandatory in undergraduate medical education. He was responsible for the postgraduate psychiatric training of large numbers of junior doctors, for many of whom he arranged further training in the UK, especially with Professor David Goldberg in the Department of Psychiatry at the University of Manchester, with whom he developed a special link. When they achieved consultant status, many of his former trainees would put up a photograph of Professor Mubasshar in a prominent position in their departments. He was also active in obtaining legal rights for people with mental illness in Pakistan. After years of incessant effort, he was instrumental in the passage of the Mental Health Ordinance 2001. His interest in promoting public policy in favour of the mentally ill more generally motivated him to make links with whoever was President of Pakistan at the time.

His influence in psychiatry extended well beyond national boundaries. His Institute of Psychiatry in Rawalpindi was recognised as a World Health Organization (WHO) Collaborating Centre for Mental Health Research and Training, of which he was the Director. Among many other international positions, he was chair of the Global Mental Health Network, Global Forum for Health Research at WHO, Geneva. The WHO Community Mental Health Programme was his brainchild, and he had a particularly influential link with the development of such programmes in the WHO Eastern Mediterranean region.

Malik Hussain Mubbashar was born in Lahore on 1 August 1945, the son of Miraj-ud-din Malik and Shahzadi Anwar. His father was a government employee in the irrigation department. After completing his medical training at King Edward Medical College, Lahore, in 1968 with honours, he underwent further medical and then psychiatric training in London at Guy's Hospital with David Stafford-Clark. While in England he passed the membership examinations of three UK medical colleges. He was strongly motivated by having seen in childhood the ill-treatment of the mentally ill.

On return to Pakistan in 1972, he was appointed to the Central Government Hospital, Rawalpindi. However, to his disappointment, the administration of the hospital made it abundantly clear that he was not welcome as a psychiatrist and refused him any clinic space to see psychiatric patients. Manifesting his inevitable grit, as he did throughout his career, he set up his table and chair under the shade of a tree in a neglected, remote corner of the hospital. Within 8 years, his department had grown into an Institute and then into a WHO Collaborating Centre.

His administrative talents and leadership qualities were rapidly recognised. Professor Mubbashar was closely involved with the College of Physicians and Surgeons Pakistan (CPSP), becoming Dean of the newly formed Faculty of Psychiatry. He was elected President of the Pakistan Psychiatric Society. He served as Principal of the Rawalpindi Medical College before going on to become the Vice-Chancellor of the University of Health Sciences, Lahore.

During his lifetime he was accorded many honours. Two of the highest civilian awards of the Pakistani government, Hilal-e-Imtiaz and Sitara-e-Imtiaz, were conferred on him. The CPSP's gold medal for psychiatry, the most prestigious medal in the field, is named the Malik Hussain Mubbashar Gold Medal.

In his spare time, he enjoyed visiting rural areas and trekking.

Professor Mubasshar is survived by his wife Dr Yasmin Mubbashar, whom he married in 1973, children Sabooh, Aamna, Zainab, Saima, Fatima, Maryam and Imtiaz (five of whom are psychiatrists) and 15 grandchildren.

